# The Carbon Cycle and Hurricanes in the United States between 1900 and 2011

**DOI:** 10.1038/srep05197

**Published:** 2014-06-06

**Authors:** Devendra Dahal, Shuguang Liu, Jennifer Oeding

**Affiliations:** 1 SGT Inc., contractor to the US Geological Survey (USGS) Earth Resources Observation and Science (EROS) Center, Sioux Falls, SD 57198; 2 US Geological Survey (USGS) Earth Resources Observation and Science (EROS) Center Sioux Falls, SD 57198

## Abstract

Hurricanes cause severe impacts on forest ecosystems in the United States. These events can substantially alter the carbon biogeochemical cycle at local to regional scales. We selected all tropical storms and more severe events that made U.S. landfall between 1900 and 2011 and used hurricane best track database, a meteorological model (HURRECON), National Land Cover Database (NLCD), U. S. Department of Agirculture Forest Service biomass dataset, and pre- and post-MODIS data to quantify individual event and annual biomass mortality. Our estimates show an average of 18.2 TgC/yr of live biomass mortality for 1900–2011 in the US with strong spatial and inter-annual variability. Results show Hurricane Camille in 1969 caused the highest aboveground biomass mortality with 59.5 TgC. Similarly 1954 had the highest annual mortality with 68.4 TgC attributed to landfalling hurricanes. The results presented are deemed useful to further investigate historical events, and the methods outlined are potentially beneficial to quantify biomass loss in future events.

Hurricanes in the North Atlantic, are major natural disturbances that are part of life in the southern and eastern United States causing severe impacts on forest ecosystems in the region^1^,^2^,^3^. The intensity of impact to forest stands is often highly variable, ranging from short-term to long-term. Short-term impacts may include defoliation and branch break or total blowdowns. Long-term impacts may include rapid or slowed tree mortality, changes in successional direction, increased species turnover and age class diversity, and faster biomass allocation and carbon storage^1^,^4^,^5^,^6^. Alterations to forest composition and structure, and transfer from live trees to dead carbon pools are certain regardless of the inter- and intra-annual frequency and intensity of hurricanes^2^,^7^,^8^,^9^. Several studies have suggested that the frequency of hurricanes would decrease but their intensity may increase in some locations as global climate warms in the future^8^,^10^. The increased hurricane intensity would certainly destroy more forest biomass whenever they pass over the forestland.

Intense hurricanes can cause substantial damage to a forest but their size alone is not enough to determine how much live tree biomass results in as debris. Forest types, wind speed, and heavy rainfall followed by flooding are the key factors for most severe storm damage^11^ to forested areas. Evergreen forests are generally more susceptible to hurricane damage than deciduous forests. Susceptibility of forest type to hurricane-related damages also varies for different types of damage, including breakage, and uprooting^12^,^13^,^14^. In addition, trees planted outside their natural range may be more susceptible to damage^15^. Immediate damage to hardwood forests range from defoliation to total blow down; however, many species may recover quickly with releafing and sprouting often occurring within 3–4 weeks^16^.

The role of individual tree mortality through its disturbance on regional carbon balance is complicated by the strong spatial and temporal nature of slow-in and rapid-out of carbon flux^17^,^18^. Thus, it is important to understand the spatiotemporal variation of disturbance and its effects on forests and carbon dynamics to quantify current mortality and to predict future change^17^. Given the variations in direction, duration, and distance of wind speed relative to the eye of the storm, predicting forest mortality from hurricane events is difficult. Different responsive natures of different tree species, age classes, soil profiles, as well as topography add to the difficulty^3^.

As the North Atlantic basin has experienced an increase in hurricane activities with some intense events in the recent past, various efforts were undertaken to quantify carbon dynamics from these activities. Some studies were primarily targeted to study biomass damage from individual events, whereas others were involved on extended spatial and temporal scales^2^,^6^,^19^,^20^. Chambers, et al.^19^ used Landsat data, field-based information, and an empirical model to quantify biomass loss from Hurricane Katrina. Zeng, et al.^2^ applied similar approaches with the addition of simulated wind field for each hurricane event to model biomass loss and carbon release from 1851 to 2000 for all US landfalling hurricanes. Nielsen^6^ used Moderate Resolution Imaging Spectroradiometer (MODIS) data, and environmental and meteorological information to predict the level of damage to forests in the Katrina impact region in the states of Louisiana and Mississippi. McNulty^11^ combined historical and current forest damage and hurricane information, and analyzed loss of forest biomass through hurricane events using timber loss vis-à-vis the proportion to leaves, roots, and stems. After Hurricane Andrew, various studies were conducted to evaluate biomass damage in different forest stands from Florida to the Gulf coast; however, none of these studies focused specifically on quantifying carbon from the biomass loss^16^,^21^,^22^. These studies recognize the important role that hurricanes play in forest biomass mortality and carbon cycles. However, none of this previous work proposed a simple approach to quantify biomass loss from individual hurricane events or produced a live biomass mortality dataset for large number of historic hurricane events. We propose a simple method to quantify live biomass mortality datasets for individual hurricane events as well as annual mortality from hurricanes using hurricane best-track data, and the National Land Cover Database (NLCD) as inputs.

This study covers hurricane events that originated in the tropical region of the Atlantic Ocean and made landfall in the conterminous United States (CONUS). The US hurricane impact zone includes the CONUS coastal region, ranging from the Gulf of Mexico to the Florida peninsula and along the Atlantic coast. Twenty-five states in the region, including the Washington DC area, have experienced impacts from historical hurricane events (Figure 1).

## Results

### Biomass mortality by individual hurricane events

Hurricane Camille, classified as a category 5 hurricane on the Saffir-Simpson scale that struck the coasts of Louisiana and Mississippi in 1969, causing 59.49 TgC in mortality of aboveground live biomass (AGB), the highest amount in US history. The other most destructive hurricane were Donna in 1960 with 51.48 TgC, Hazel in 1954 with 47.39 TgC, and Okeechobee in 1928 with 41.22 TgC (informally named after the event^23^). These events were classified as category 4 hurricanes when they made landfall (Table 1). These hurricanes caused high biomass mortality because they were passing over largely forested areas. Only 10% of the 330 hurricane events in the study period caused ≥30 TgC biomass mortality, whereas, nearly 60% of these hurricane events destroyed ≤5 TgC (Figure 2A).

### Annual biomass mortality from hurricanes

Between 1900 and 2011, the average annual loss of AGB was calculated as 18.2 TgC (the range was between 12.7 and 23.5 TgC). The highest number of hurricanes making landfall in the United States that were greater than 35 mph occurred in 1916 (nine), followed by 2004 and 2005 (eight each year); however, the highest annual AGB loss was highest in 1954 with 68.37 TgC. The three years with the highest number of landfall hurricanes (1916, 2004, and 2005) included three or more hurricanes rated as category 4 or higher. In 1954, only four hurricanes made landfall in the U.S.; three of them were rated as category 3 or higher. The other most destructive years in terms of AGB mortality were 1960 and 1969 with 65.95 TgC and 63.76 TgC, respectively (Table 2). In 1922, no hurricanes made landfall on U.S. mainland, and there were 12 years (1905, 1925, 1931, 1951, 1962, 1973, 1978, 1980, 1987, 1990, 2006, and 2010) when ≤1 TgC of AGB mortality occurred from landfalling hurricanes (Figure 3). Landfalling hurricanes accounted for more than 55 TgC/yr only during 8 years (Table 2). On the other hand, 89 years experienced less than 30 TgC/yr AGB mortality annually, while 33 years had 5 TgC/yr or less biomass destruction (Figure 2B).

### Spatial pattern of biomass mortality

**Figure 1:**
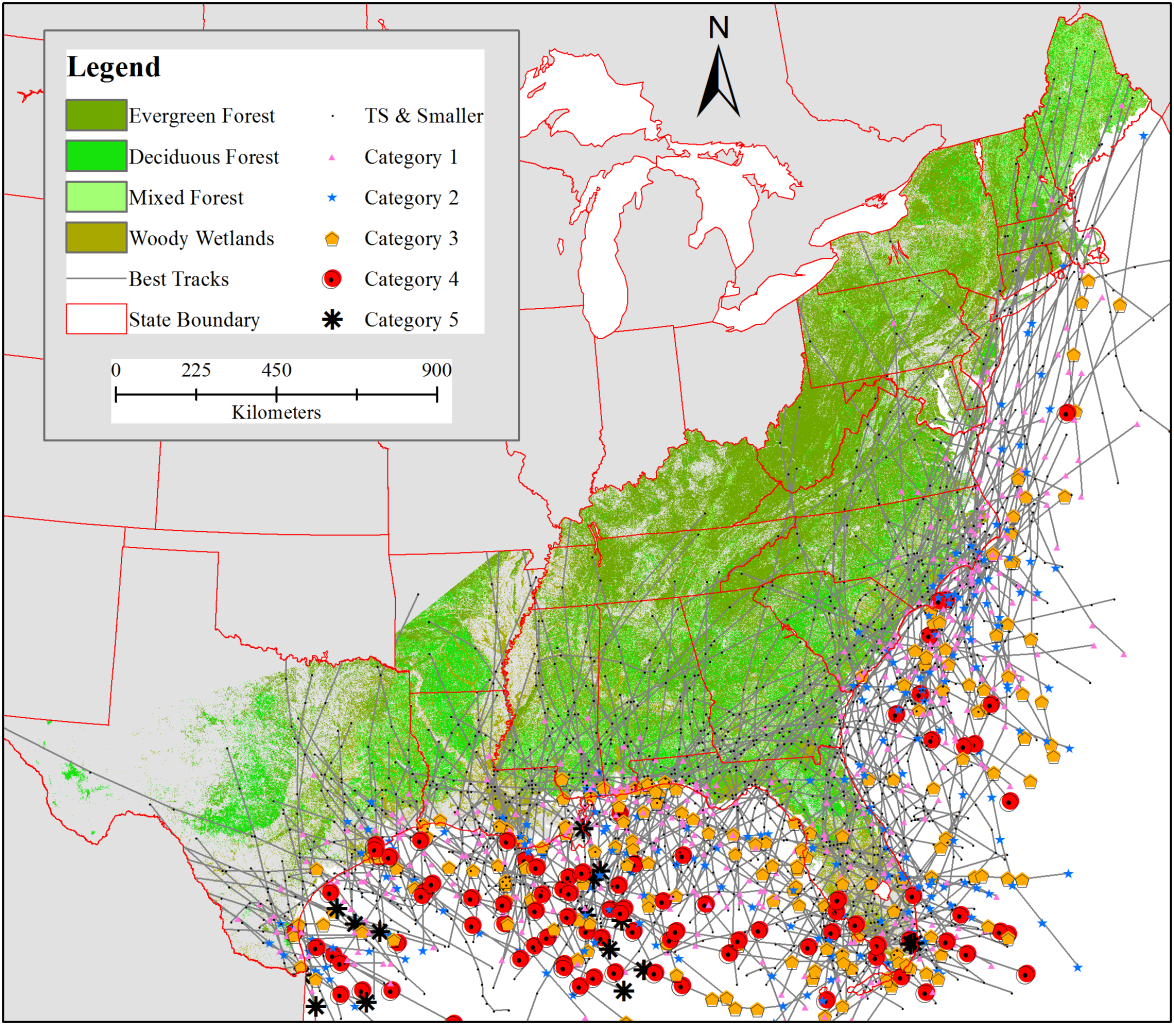
Forest areas were derived from NLCD 2001. Hurricane best tracks and category data are from HURDAT database for 1900–2011. Categories are based on the Saffir-Simpson scale and TS stands for tropical storm. Best tracks were cut off after they decreased below a sustained weed speed of 18 mph over land. Map generated using ArcGIS 10.1.

The Gulf coast (coastal region of Louisiana, Mississippi, Alabama, and northwestern Florida) and the mid-Atlantic coast (Virginia and North Carolina coastal areas) (Figure 1), lost higher biomass than any other areas on average per square meter over the studied time period (Figure 4), which coincides with weather-related forest mortality maps from Forest Inventory and Analysis (FIA) inventories^24^. Spatially, the 1925–1950 time period had less biomass mortality rate per square meter, even though hurricanes making landfalling during that period were not far from the long-term annual average (2.94 events per year)^25^,^26^. On the other hand, the periods from 1950–1975 and 1975–2000 had below annual average landfalling hurricane events (2.78 and 2.52, respectively, versus 2.94 events per year)^25^,^26^. However, these periods had above average biomass mortality rate (see Figure 3 and Figure 4) because of some highly intense hurricanes (such as Hazel 1954, Donna 1960, Camille 1969, Hugo 1989, and Elena 1985) making landfall over largely forested regions^3^.

## Discussion

The long-term annual average biomass mortality from this study (18.2 TgC/yr for 1900–2011) was comparable with the results from Zeng, et al.^2^, who estimated an average of 19.5 TgC/yr live biomass loss for the 1900–2000 period after synthesizing field measurements, performing satellite image analysis, and using some complex empirical models. Annual biomass mortality in this study was an aggregation of mortality from all events of the particular year on a pixel-by-pixel basis, and biomass mortality from individual events was estimated using some simple datasets (forest area and biomass stock) and field-measured coefficients. For example, if any location was experienced hurricane events more than once in a particular year, the maximum destruction from those events was taken into account rather than the sum of destruction from all events. As McNulty^11^ explains, this approach is more reasonable because a substantial portion of biomass destroyed by a first hurricane event would recover by the second hurricane event to hit the same area, even if the event occurred within a couple of months.

Annual average biomass loss was different for each portion of the studied time period. The largest loss was estimated for 1950–1974 with 22.6 TgC/yr (Table 3); this period included the top three most destructive hurricane events (Table 1). Overall, 2.78 hurricane events per year made landfall during the period from 1950–1974, compared to 2.94 events per year for the entire study period (1900–2011), but a higher number of these events were more intense (category 3 and above). Most intense hurricanes also passed through highly forested regions damaging a large amount of biomass because biomass mortality is dependent not only on the intensity of hurricanes but also the presence of AGB^11^. Annual average AGB mortality for the 2000–2011 period was substantially lower than the long-term annual average (15.46 versus 18.15 TgC/yr) but our estimates were similar to FIA weather-related biomass mortality data (15.5 TgC/yr) for the hurricane impact area (Table 3 and Figure 3). The FIA estimation should have a slightly higher mortality than this study because it includes all types of weather-related forest mortality (ice, wind, hailstorm, tornadoes, windstorms, and hurricanes) in a single discrete class. FIA measurements may have underestimated the actual loss because forest regeneration and succession are rapid after hurricane-like disturbances^11^,^17^. Additionally, there is temporal scale variability of FIA data collection. FIA compiles mortality data through plot inventory taken every 5 to 7 years for most states, and to qualify for any given disturbance, at least 25% of the trees should sustain damage or mortality in an acre of land since the previous visit^17^. Particularly, some states (for example, Mississippi) have participated in special accelerated inventories. Following Hurricane Katrina, the entire state of Mississippi was inventoried within a 687-day time span^27^ since the timing of the damage measurement after hurricane events is sensitive^3^,^28^.

For a long-term study such as this, the dataset availability for frequent time intervals is important because a lack of historical spatial datasets can add a significant level of uncertainty in the results. We analyzed the uncertainty using historical forest area and biomass storage data reported by Smith, et al.^29^ and Birdsey and Heath^30^. Our results appear to overestimate by up to 10%for certain years because of a lack of historical spatial forest area dataset (red line in Figure 3). Similarly, lack of historical spatial biomass stock datasets may have offset the result by up to 50% (green line in Figure 3). The combination of not having both of these datasets reveals an even greater effect (see the blue line in Figure 3). Therefore, at the very least, the availability of decadal spatial datasets of forest area and biomass storage availability may render lower uncertainty in our results.

**Figure 2:**
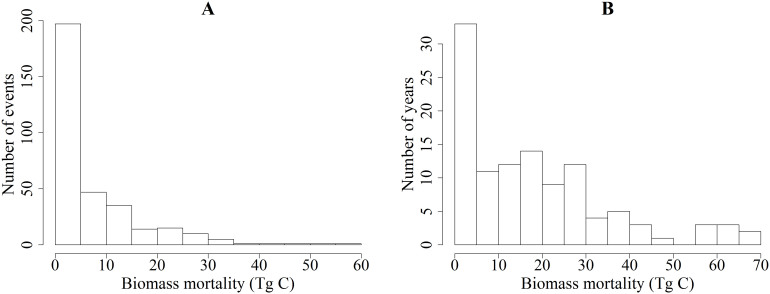
Histogram showing biomass mortality distribution by years (A) and by events (B) between 1900 and 2011.

**Figure 3:**
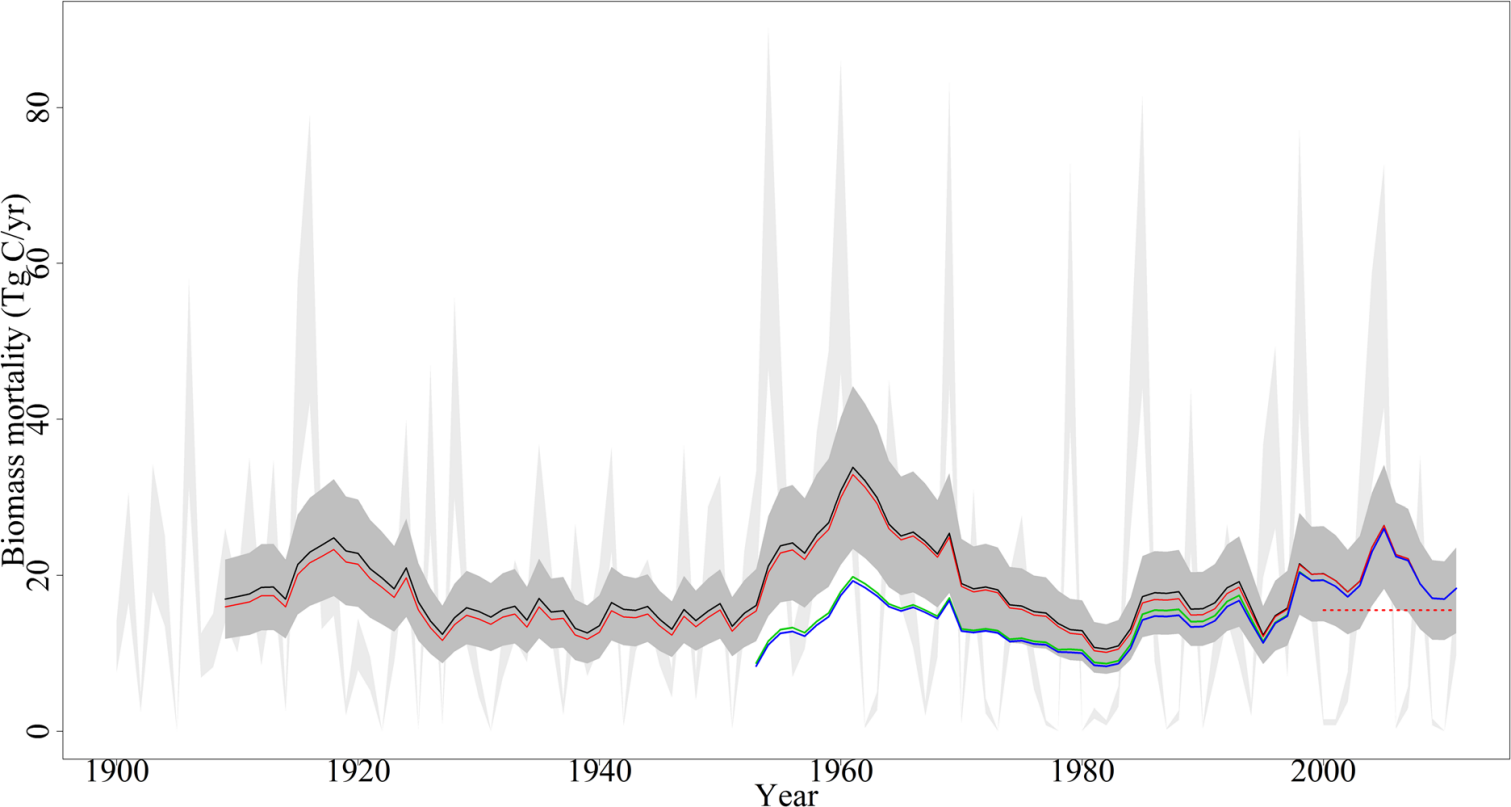
Range of annual biomass mortality (light grey shade) and range of the 10 -year average (dark grey shade) between 1900 and 2011. The mean of the 10 -year average is the black solid line, the mean of the 10 -year average adjusted with forest area is the red solid line, the mean of the 10 -year average adjusted with biomass stock is the green line, and the mean of the 10 -year average adjusted with a combination of forest area and biomass stock and FIA estimates is the red dotted line.

**Figure 4:**
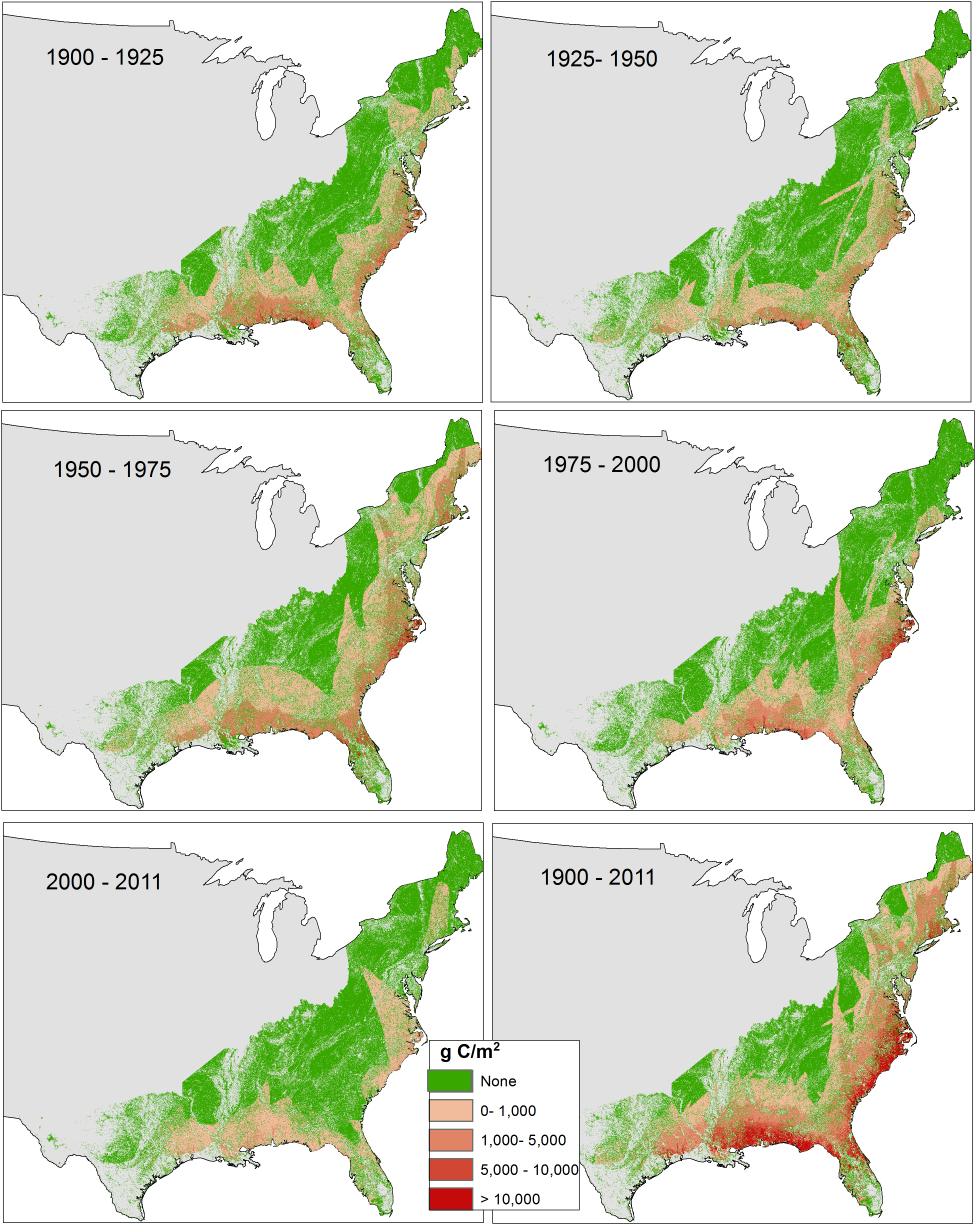
Long-term average biomass mortality from hurricane events for CONUS over different time periods. Map generated using ArcGIS 10.1.

**Figure 5:**
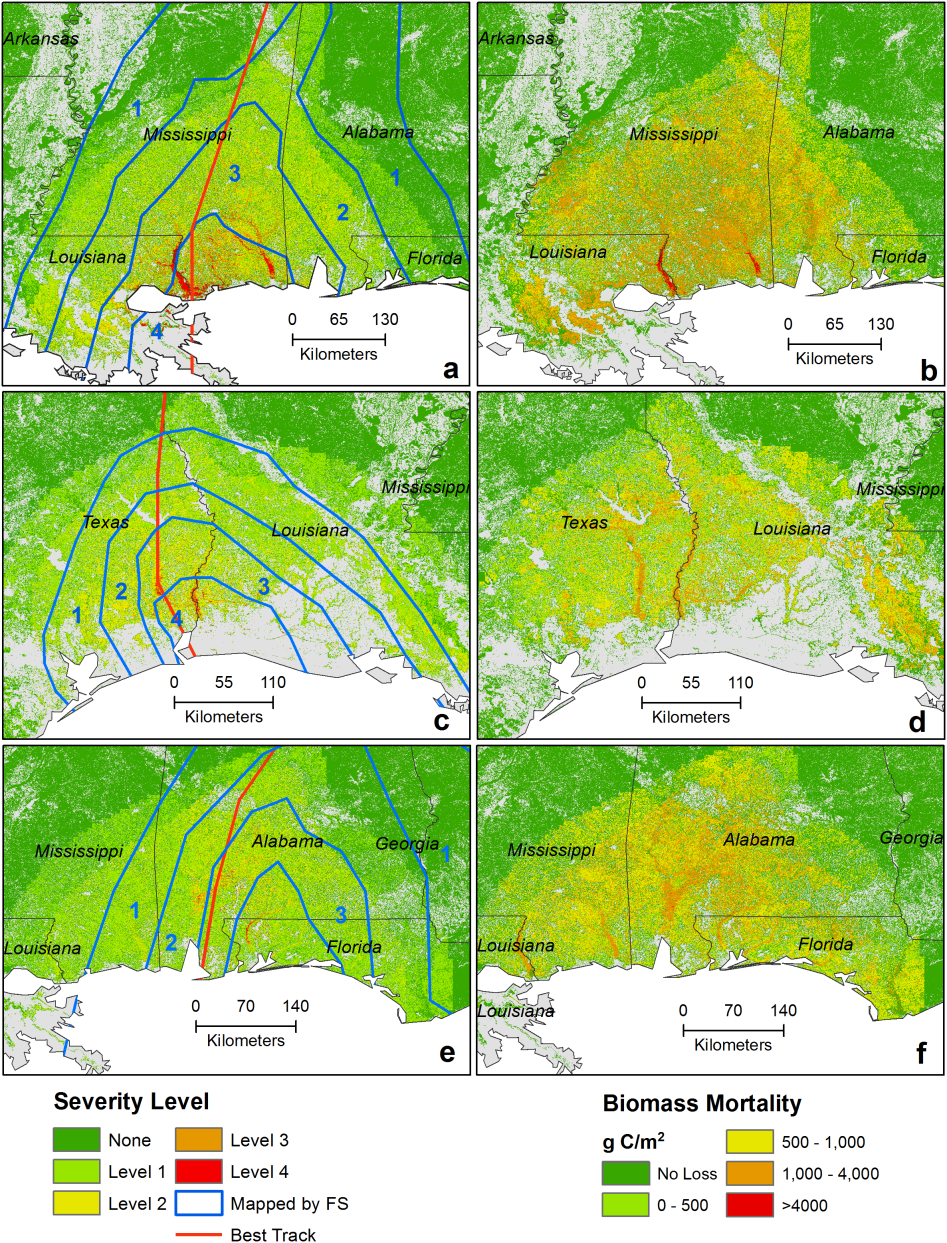
Examples of simulated severity levels and biomass mortality for three events; a, c, and e are damage severity zones of Katrina, Rita, and Ivan, respectively; b, d, and f are biomass mortality from Katrina, Rita, and Ivan, respectively. Map generated using ArcGIS 10.1.

Because the immediate effect of hurricanes in forests is conversion of live biomass to dead carbon pool, finding damaged areas after each hurricane event is critical to quantifying live biomass mortality to illustrate the distribution of biomass damage in the impact zone^6^,^31^. Mapped damage severity zones of this study for some recent events were compared with damage severity zones defined by the USDA Forest Service^32^ (Figure 2). The Forest Service established damage severity zones based on field observations of various forest sample plots. Almost always, a hurricane caused more damage on the right side of its track than the left side (see left panel of Figure 2). As Chen, et al.^33^ stated, wind and stress could be as much as 25% higher on the right side of a hurricane track than on the left side. However, the damaging distance from the track could vary from one event to another as every hurricane has a different level of wind strength, forward speed, and geographic size^34^, as well as various components that could affect the hurricane's course and intensity^33^,^35^. This study also found that every hurricane event had a different footprint of damage area and level of biomass mortality but the right side of the tracks consistently had more impact (see some examples in Figure 5).

Hurricane Katrina was one of the most widely studied hurricane events that landed in the United States. FIA mapped a higher severe zone on the right side of the track^32^ of Katrina, which coincides with this study (Figure 5a). This study also found most of the forest biomass damage occurred on the right side of the track (Figure 5b). Our study estimated that total biomass destruction by Hurricane Katrina was 36 TgC (ranging from 27.8 to 46.3 TgC), which is comparable to the results of Negrón-Juárez, et al.^7^, who estimated 43.9 TgC (±8.4 TgC) using a relationship between field-measured tree mortality and Landsat data. On the other hand, the spatial map developed by Chambers, et al.^19^ was comparable to this study (with Figure 5b) but their estimation of total biomass loss from Hurricane Katrina was 102 TgC. Chambers, et al.^19^ included surface litter, whereas this study and the study by Negrón-Juárez, et al.^7^ did not, and yet, their results appear to overestimate the total biomass loss because it is unlikely that surface litter accounts for more than twice the aboveground live forest biomass^36^,^37^.

The estimated impacts of hurricanes on carbon dynamics are highly dependent on the linkage between remotely sensed signals and ground observation of tree damage (see Table 3). Often different field-based studies suggest different damage levels even for the same event. For example, Nielsen^6^ reported that Hurricane Katrina's severity of forest damage ranged from 15 to more than 50%. Stanturf, et al.^3^ pointed out that Hurricane Katrina destroyed approximately 67% of the trees in the severely damaged zones and 33–66% and 3–33% in moderately, and lightly damaged zones, respectively. An inventory performed by the Forest Service in the DeSoto National Forest after Hurricane Katrina shows up to 83% tree damage per acre in severely damaged plots^38^. The forest damage and severity zones are also influenced by tree species. For example, over 80% of all pines were overtly damaged by Hurricane Andrew in the Everglades of southern Florida^16^,^21^. After Hurricane Andrew, about 85% of hardwood hammocks were in some way affected but average tree mortality was only 11.5% when assessed after 4 months following the event in the Long Pine Key, Everglades National Park^16^,^22^. Conducing additional field studies to systematically cover more events and locations should improve the estimates on the biomass loss percentage for each severity class and reduce overall uncertainty.

Although our approach provides spatially explicit estimates of biomass conversion from live to dead carbon pool after storms, it has some drawbacks. Uncertainty in the biomass loss such as salvaging after hurricane events was not considered in this study but that could make a significant difference in the values presented. As Stanturf, et al.^3^ noted, 37% of damaged biomass was salvaged in South Carolina following Hurricane Hugo in 1989. Our approach can only quantify immediate biomass mortality, yet many tree species tend to die many months and even years after hurricane events^3^,^11^. In addition, we did not consider the impacts of climate and land cover change on carbon pools in the reconstruction of the long-term historical carbon loss, which might have overestimated the carbon loss especially in the early time periods when forests were young and recovering from agricultural abandonment^39^,^40^.

## Methods

### Characterization of hurricanes

The best tracks for individual hurricanes that had wind speeds of at least 35 mph at landfall were retrieved from the National Hurricane Center (NHC) data archive^41^. NHC maintains hurricane track datasets beginning in 1851 with latitude, longitude, minimum pressure, and maximum wind speed at the center of circulation documented every 6 hours. The best track information is used to simulate wind field with 5-km pixel size using the HURRECON model. HURRECON is a meteorological model that can simulate wind field for a user-defined resolution using maximum wind speed, hurricane radius, and geographical locations of the hurricane's eyes. The hurricane track data must contain maximum wind speeds and geographical locations of the hurricane's centers^1^,^2^,^4^ whereas hurricane radius is calculated using the wind speed and geographical location of the hurricane's eyes^42^.

### Remote sensing of storm damage

The MODIS Reflectance product MCD43A4, which is adjusted to nadir using a bidirectional reflectance distribution function (BRDF), contains visible and infrared surface reflectance at 500 m resolution for each 16-day period. This product is generated from both Terra and Aqua data to provide the highest probability for quality input data. Atmospheric effects have been removed from this product as it would have been measured at ground level, so this is more consistent for observing vegetation status than other MODIS reflectance products^31^,^43^,^44^. The MODIS Reflectance products were used to derive Normalized Difference of Infrared Index (NDII) because of its sensitivity to vegetation water content or weight of water per unit area^31^,^45^ and better estimation of vegetation disturbance after hurricanes^31^. NDII is calculated 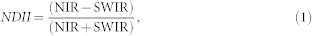
where shortwave infrared (SWIR) at 1.6–2.13 μm wavelength and near infrared (NIR) at 0.86 μm wavelength were used. MODIS products have two bands with SWIR spectra, however we adopted the 2.13 μm band due to the large amount of missing observations caused by a serious striping issue in the MODIS Aqua 1.65 μm band^31^.

To estimate biomass damage, we calculated differences of pre- and post-NDII of hurricane events as: 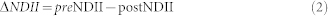
We calculated average and standard deviation of ΔNDII for the land area that surpassed a wind speed of 35 mph – values that we could use in subsequent calculations.

### Calculating the impacts of storms on tree mortality

This study followed the FIA defined naming convention for forest damage zones to compute severity classes. FIA conducts ground inventory to estimate forest damage for each plot as the percentage of basal area that suffered no damage, scattered light (Level 1), light (Level 2), moderate (Level 3) or severe damage (Level 4)^31^,^46^,^47^. The percentage of biomass loss for each severity class in this study was developed by summarizing results from various studies (Table 4). We applied bootstrap sampling^48^ with 10 iterations to calculate the mean and standard deviation of means in order to quantify the range of biomass loss for each event.

**Figure 6:**
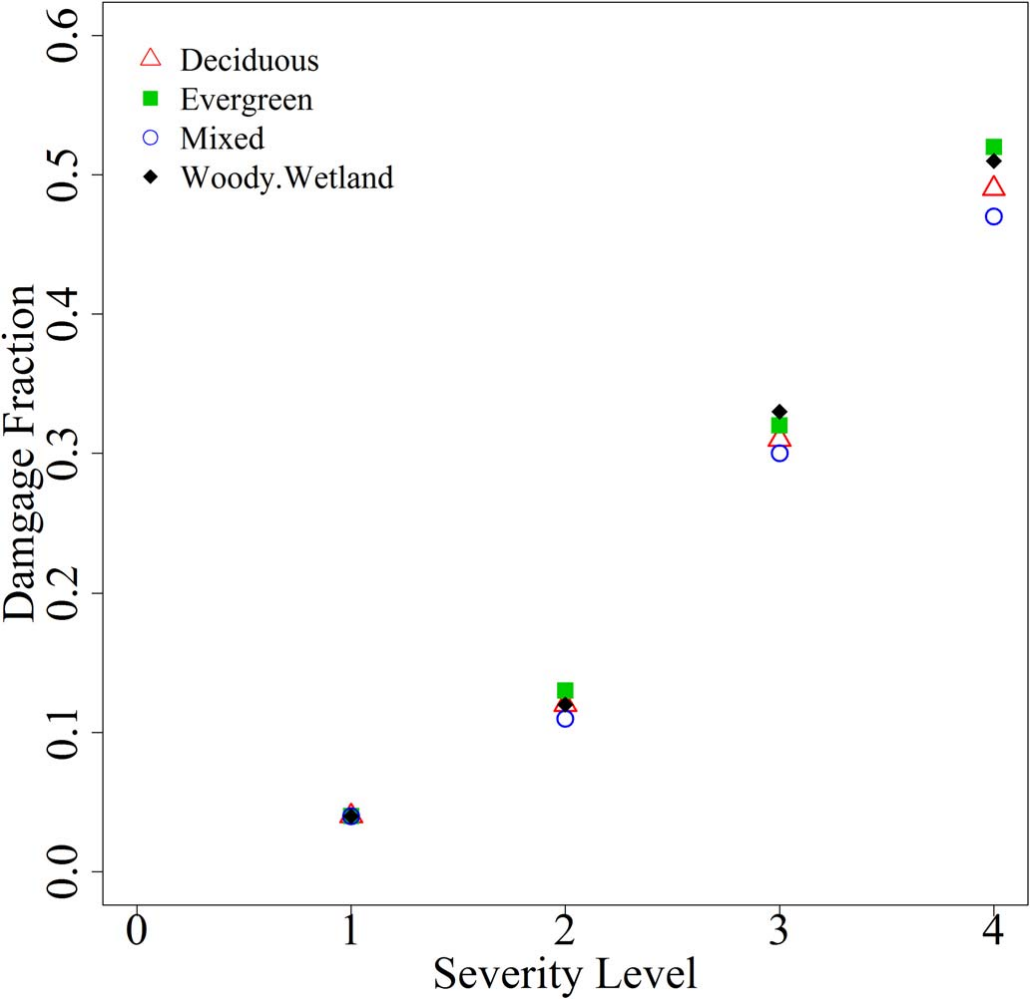
Average value of damage fraction for forest type by severity level.

**Figure 7:**
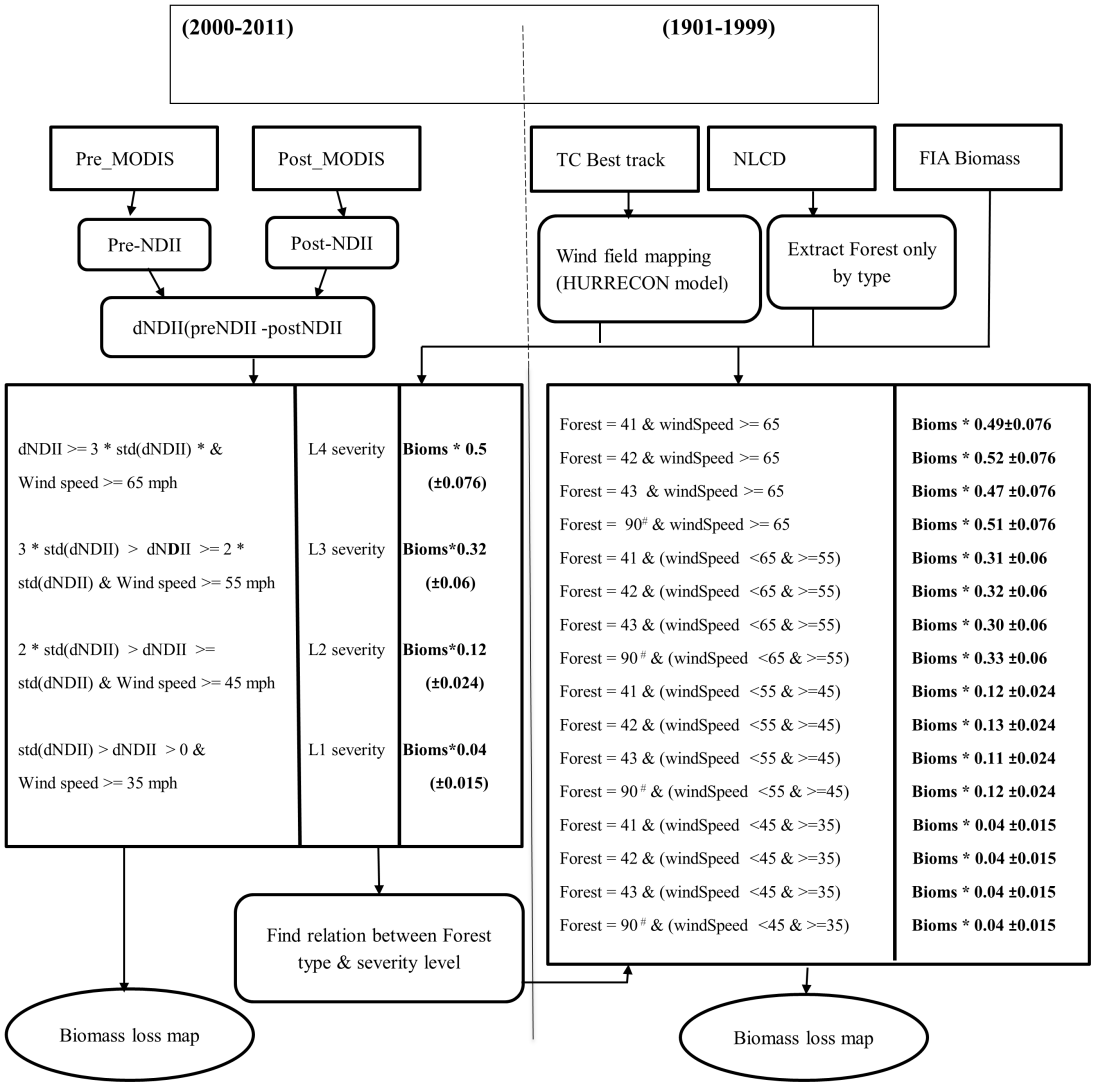
Flowchart showing methodology applied in this study; the left panel depicts the remote sensing approach and the right panel shows the forest type damage fraction approach. Forest types 41, 42, 43, and 90 are deciduous, evergreen, mixed, and woody wetland respectively; forest type for woody wetlands for NLCD 1992 is 91.

We employed a remote sensing approach (i.e., using MODIS) to estimate the range of biomass damage from 2000 to 2011 using average and standard deviation values of damage fraction presented in Table 4. Because MODIS data were not available before 2000, we had to develop a reliable damage fraction for different forest types. As Stanturf, et al.^3^ and Vanderwel, et al.^17^ noted, different forest species and types respond differently to various kinds of disturbances. We used individual damage severity maps developed through a remote sensing approach from 2000–2011, and extracted the biomass damage for each forest type for individual events. We further calculated percentage of biomass damage by forest type to establish a relationship between forest types and severity level of biomass damage (Figure 6). The percentage of biomass damage by forest type from individual events was then averaged and applied for quantifying biomass damage for years between 1900 and 1999 (see Figure 7); we named this approach *forest type damage fraction*.

### Land cover and biomass storage

NLCD inputs for 1992, 2001 and 2006 were retrieved from http://www.mrlc.gov/finddata.php. We masked out forestland from the NLCD datasets for the hurricane impact region in the CONUS. NLCD 1992 was used to derive forest pixels prior to 1995, NLCD 2001 was used for the period 1996–2005, and NLCD 2006 was used for the post-2006 time period. Woody wetland was also included in this study as forested land with three forest types: deciduous, evergreen, and mixed.

CONUS forest live biomass data were downloaded from http://fsgeodata.fs.fed.us/rastergateway/biomass. The forest live biomass dataset was validated with FIA field-measured data for randomly selected plots from 65 CONUS mapping zones. The correlation coefficient of the observed field-measured data and the live biomass datasets ranged between 0.79 and 0.31 among mapping zones^49^. The unit of the dataset is converted to carbon equivalent by multiplying by 0.5^2^,^50^.

### Uncertainty analysis

Given the lack of historical spatial datasets to map forest cover and biomass prior to 1992, we postulate that a propagation of uncertainty was introduced. To map the uncertainty, we took historical total forest area and biomass stock, and calculated the ratio of changes from the base year of 2000 reported by Smith, et al. 29 and Birdsey and Heath 30. The periodic coefficient values were then interpolated to develop annual values, which were then multiplied to annual biomass mortality to derive adjusted values.

We assume that coefficient values for severity classes also introduced some uncertainties as these values were derived from smaller pool of literature and no climatic variables were considered in this study. The results would have been different if more literature values were available and climatic variables were incorporated, however, this is work for future research.

## Author Contributions

S.L. and D.D. designed the research. D.D. performed research and wrote the manuscript under the guidance of S.L. J.O. contributed on collecting data and writing up the manuscript. All authors reviewed the manuscript.

## Figures and Tables

**Table 1:** Ten most destructive hurricane events with dates and categories at landfalling. Numbers in parentheses show minimum and maximum range

Name	Landfalling Date	Category at Landfalling*	Biomass mortality (TgC)
Camille	8/15/1969	5	59.49 (41.42 ~ 77.58)
Donna	9/10/1960	4	51.48 (35.80 ~ 67.16)
Hazel	10/15/1954	4	47.39 (32.27 ~ 62.51)
Okeechobee	9/17/1928	4	41.22 (28.78 ~ 53.69)
Elena	9/1/1985	3	38.42 (26.98 ~ 49.86)
Katrina	8/29/2005	3	36.03 (27.75 ~ 46.29)
Gracie	9/29/1959	3	33.85 (23.47 ~ 44.21)
Diana	9/13/1984	3	33.43 (23.18 ~ 43.68)
Hugo	9/22/1989	4	30.47 (20.91 ~ 40.05)
Frederic	9/13/1979	3	30.4 (21.22 ~ 39.58)

*based on Saffir-Simpson scale.

**Table 2:** Ten most destructive years and number of hurricane events with biomass mortality values. Numbers in parentheses show the minimum and maximum range

Year	Number of Events	Category > = 3	Biomass mortality (TgC)
1954	4	3	68.37 (46.57 ~ 90.18)
1960	4	1	65.95 (45.98 ~ 85.93)
1969	3	1	63.76 (44.38 ~ 83.18)
1985	6	1	62.66 (43.94 ~ 81.38)
1916	9	1	60.59 (42.18 ~ 78.99)
1998	5	0	59.17 (41.23 ~ 77.11)
2005	8	4	56.42 (41.56 ~ 72.61)
1979	5	1	55.86 (38.92 ~ 72.8)
2004	8	3	46.24 (31.52 ~ 58.81)
1906	4	1	44.59 (31.23 ~ 57.96)

**Table 3:** Annual average biomass mortality divided into 25-year periods plus the last 12 years of the study period. Numbers in parentheses show the range of minimum and maximum values

Time	Biomass Mortality (TgC/yr)
1900–1924	18.49 (12.93 ~ 24.05)
1925–1949	15.04 (10.63 ~ 19.30)
1950–1974	22.61 (15.72 ~ 29.49)
1975–1999	17.75 (12.37 ~ 23.12)
2000–2011	15.46 (10.59 ~ 19.90)
*1900*–*2011*	*18.15 (12.66* ~ *23.5)*

**Table 4:** Summary of percentage values of biomass loss for each severity class from various studies

Hurricane event	Level 1	Level 2	Level 3	Level 4	Source
Katrina 2005	10	15	-	50	Nielsen^6^
Katrina 2005	3	18	49.5	67	Stanturf, et al.^3^
Katrina 2005	-	11.5	22.5	40	Meeker, et al.^38^
Katrina 2005	1.17	1.36	10.47	30.91	Oswalt and Oswalt^51^
Rita 2005	3	15	50	60	*Texas Forest Service*^46^
Ike 2008	3	10	25	-	*Texas Forest Service*^47^
**Average**	**4.03**	**11.81**	**31.49**	**49.58**	
**STD of Means**	**1.45**	**2.44**	**6.01**	**7.58**	
